# Monensin intoxication due to intraruminal boluses in beef cattle: retrospective analysis of 7 outbreaks

**DOI:** 10.29374/2527-2179.bjvm006525

**Published:** 2025-11-10

**Authors:** Agustina Tettamanti, Germán José Cantón, Emiliano Sosa, Eleonora Morrell, María Valeria Scioli, Delfina Balbuena, Juan Agustín García

**Affiliations:** 1 Instituto de Innovación para la Producción Agropecuaria y el Desarrollo Sostenible (IPADS), INTA Balcarce-CONICET, Balcarce 7620, Buenos Aires, Argentina.

**Keywords:** myotoxic, cardiomyopathy, poisoning, ionophores, ruminants, miotóxico, cardiomiopatia, intoxicação, ionóforos, ruminantes

## Abstract

Monensin is an ionophore antibiotic well known for its multiple benefits in cattle diets. Toxic monensin Lethal Dose 50 (LD_50_) concentrations in cattle are reported from 26 mg/kg BW. Intraruminal monensin boluses consist of controlled-released capsules, often administered to beef or dairy cattle. Seven outbreaks were analyzed, all occurring in Buenos Aires province between 1997 and 2024 affecting steers in extensive or semi-extensive grazing systems. Clinical signs appeared one to five months after boluses application and included loss of appetite, decay, recumbency, reluctance to move, tachypnea, isolation, muscle tremors, ear drooping and sudden death. Twelve necropsies were performed, and gross findings included pale multifocal streaks myocardium of both ventricles, edema in cavities and mesenteries, hepatomegaly, and diffusely non collapsed or inflated lung with interlobular edema. Microscopically, the most characteristic lesion present in all 12 necropsies was necrotizing cardiomyopathy in different stages of regeneration, some of them being polyphasic. Masson trichrome stain revealed fibrous connective tissue in extensive multiple areas replacing cardiac muscle cells. Mean serum CPK was considered moderately to highly elevated. Herein in all 7 outbreaks, boluses apparently failed to release pills correctly. In conclusion, though toxicosis after failure of intraruminal boluses in grazing cattle are rare, it is important to describe the occurrence of cases when release bolus mechanism fails, causing intoxication that evolves from subacute to chronic, with characteristic pathological findings of polyphasic muscle lesions.

## Resumo

A monensina é um antibiótico ionóforo amplamente conhecido por seus múltiplos benefícios em dietas de bovinos. Concentrações tóxicas de monensina com DL50 em bovinos são relatadas a partir de 26 mg/kg de peso corporal. Os bolos intraruminais de monensina consistem em cápsulas de liberação controlada, frequentemente administradas a bovinos de corte ou leiteiros. Foram analisados sete surtos, todos ocorridos na província de Buenos Aires entre 1997 e 2024, afetando novilhos em sistemas de pastagem extensivos ou semi-extensivos. Os sinais clínicos surgiram de um a cinco meses após a aplicação dos bolos e incluíram perda de apetite, apatia, decúbito, relutância em se mover, taquipneia, isolamento, tremores musculares, orelhas caídas e morte súbita. Foram realizadas doze necropsias, revelando lesões macroscópicas como estrias multifocais pálidas ao longo do miocárdio de ambos os ventrículos, edema em cavidades e mesentérios, ígado aumentado com bordas arredondadas e padrão lobular marcante de cor castanho-claro a castanho-avermelhado e pulmões aumentados de forma difusa com edema interlobular. Microscopicamente, a lesão mais característica presente nas 12 autópsias foi a cardiomiopatia necrosante em diferentes estágios de regeneração, algumas sendo polifásicas. A coloração de tricrômico de Masson revelou tecido conjuntivo fibroso substituindo células musculares cardíacas em áreas extensas e múltiplas. Os níveis médios de CPK sérico foram considerados moderada a altamente elevados. Em todos os 7 surtos, os bólus aparentemente falharam na liberação correta das pílulas. Conclui-se que, embora a toxicoses por falha nos bolos intraruminais em bovinos a pasto sejam raras, é importante descrever a ocorrência de casos em que o mecanismo de liberação falha, levando a quadros patológicos subagudos a crônicos com lesões musculares polifásicas.

## Introduction

Monensin is an ionophore antibiotic produced from the fungus *Streptomyces cinnamonensis* (Basaraba et al., 1999; Ensley, 2020). It is well known for its multiple benefits in cattle diets, including improved feed conversion efficiency, prevention of bloat and acidosis, coccidiosis control, among others (Brito et al., 2020; Callaway et al., 2003, Ensley, 2020). Episodes of monensin intoxication may occur due to supplementation errors when administered as feed additive (Brito et al., 2020; Gabor & Downing, 2003; Gonzalez et al., 2005; Varga & Puschner, 2012). Monensin intoxication can also occur when used in other ruminant species with different susceptibility, as previously reported in sheep (Romero et al., 2018), goat (Anjos et al., 2023) or water buffaloes (Bence et al., 2018; Rozza et al., 2006). Accidental poisoning in dogs after chewing intraruminal monensin boluses expelled by cows is also reported (Condon & McKenzie, 2002). Ionophores mechanism of action is related to formation of lipid-soluble complexes and transport of ions across biologic membranes; particularly monensin mediates sodium and hydrogen exchange causing cellular damage through alteration of intracellular ionic homeostasis with massive influx of sodium and calcium cytoplasmic increase into muscle cells during overdose, destabilizing biological membranes, with consequent degeneration and necrosis of cardiac and skeletal muscle cells (Brito et al., 2020; Gabor & Downing, 2003; Hall, 2004). Toxic monensin LD50 concentrations in cattle are reported from 26 mg/kg BW, while therapeutic dosage range between 0,5 and 0,7 mg/kg BW (Basaraba et al., 1999; Cooper & Valentine, 2016; Duffield et al., 1999; Ensley, 2020).

Intraruminal monensin boluses consist of controlled-released capsules, often administered to beef or dairy cattle (Duffield et al., 1999; Grainger et al., 2008; Green et al., 1999). There is only one report of monensin poisoning in cattle due to the simultaneous use of intraruminal boluses and oral administration of monensin salts (Odriozola et al., 1999). To our knowledge monensin intoxication through intraruminal boluses was not previously reported in the literature. Herein we describe 7 outbreaks of intoxication associated with monensin intraruminal boluses in beef cattle reviewing epidemiological data and clinicopathological features with emphasize in pathological differences compared to more frequent acute monensin intoxication. Prolonged exposure to a monensin overdose, in small doses, likely accounts for the extended clinical course and the eventual development of more noticeable gross myocardial lesions.

## Case description

**Figure 1:**
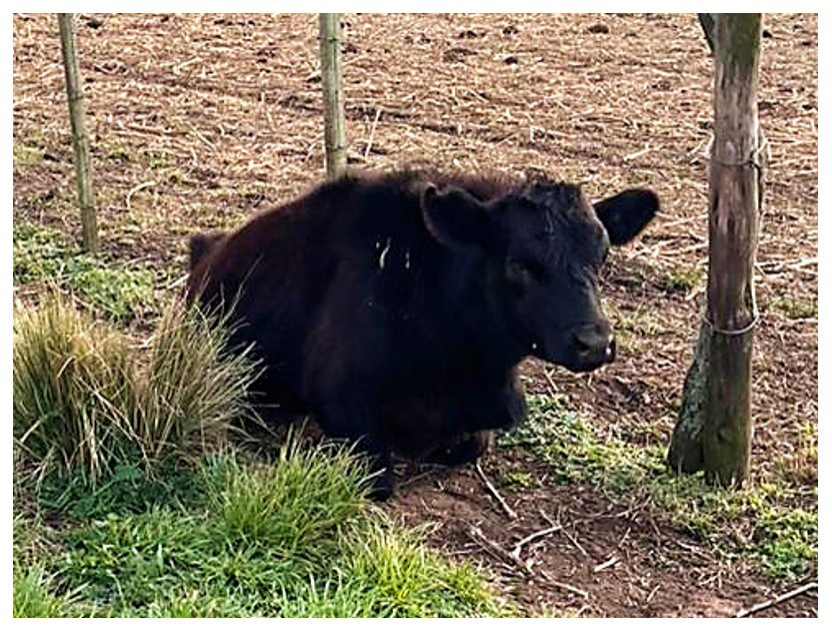
Steer with monensin intoxication due to intraruminal boluses, exhibiting loss of sternal recumbency, reluctance to incorporate and move and isolated from the herd.

Eighty-five outbreaks of monensin intoxication in cattle were registered by the Specialized Veterinary Diagnostic Service (SDVE) INTA Balcarce between 1997 and 2024. Seven (8.23%) were associated with intra-ruminal boluses administration. Clinical and epidemiological data were collected, including geographical location, month of outbreak occurrence and boluses placement, affected cattle category, grazing source, clinical signs, and incidence rates. The 7 outbreaks occurred in Buenos Aires province, Argentina, between 1997 and 2024, in beef farms (Table 1). All outbreaks occurred in semi-extensive grazing systems, including Aberdeen Angus and crossbreed steers (6/7). A mean low incidence and mortality was registered, of 1.91% (0.5% to 4%) and 1.78% (0.4% to 4%), respectively, though it resulted in a high mean lethality of 80.4% (30% to 100%). Clinical signs (Figure 1) were observed from one to five months after bolus administration, including loss of appetite, decay, recumbency, reluctance to move, tachypnea, isolation, muscle tremors, ear drooping and sudden death.

**Table 1:** Epidemiological data of 7 outbreaks of intoxication due to intraruminal monensin boluses in beef cattle.

**Year**	**Month of boluses administration**	**Month of outbreak**	**Production system**	**Diet**	**Days of first clinical signs after bolus administration**
1997	NR	December	Semi-extensive	Pasture-based forage	NR
2002	November	December	NR	NR	30
2002	October	December	Semi-extensive	Pasture-based forage	60
2003	NR	October	NR	NR	NR
2004	October	December	NR	NR	60
2022	August	September	Semi-extensive	Pasture-based forage and deferred corn	30
2024	March	August	Semi-extensive	Pasture-based forage, hay and corn.	150

NR: not registered.

**Figure 2:**
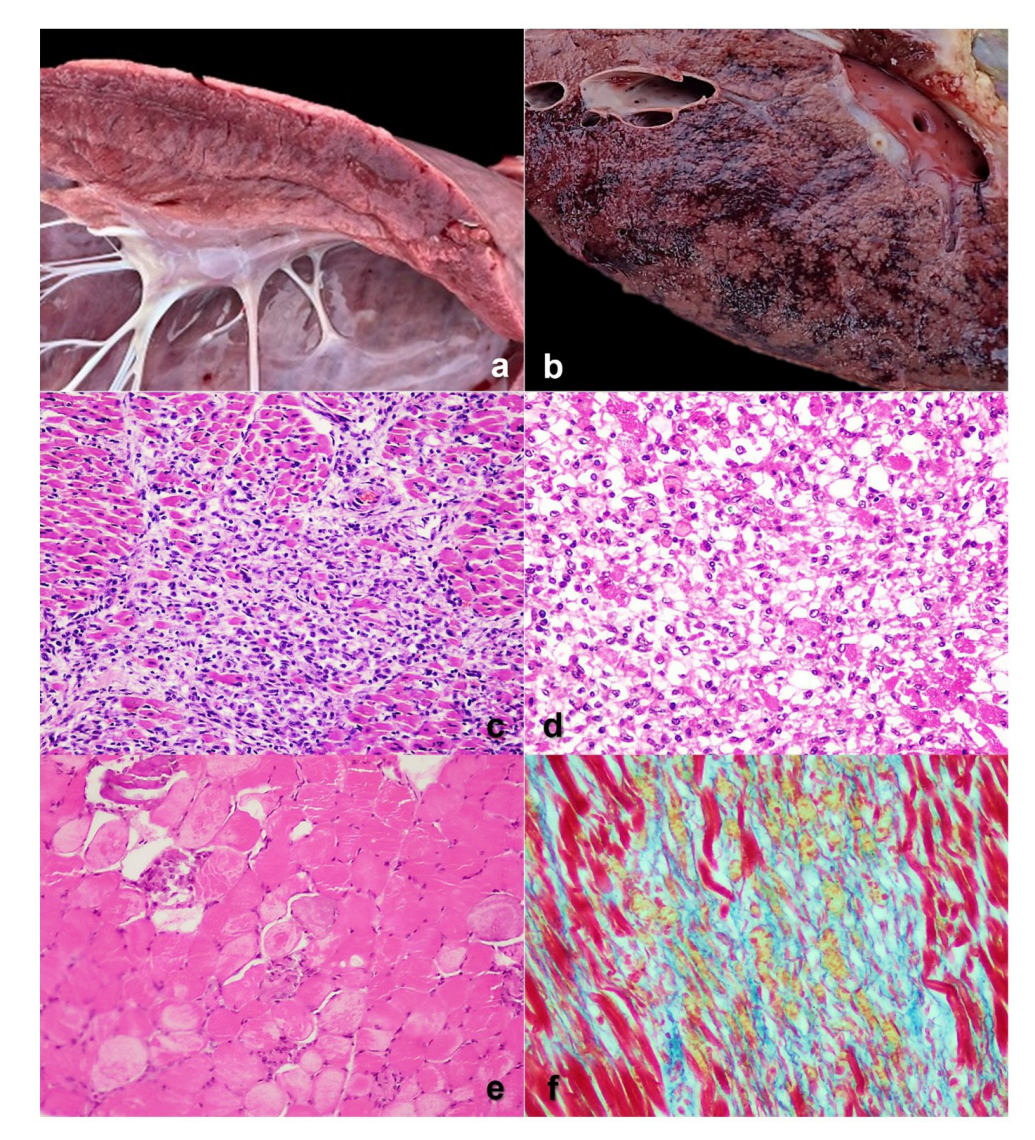
Main gross (A and B) and microscopic (C to F) findings in steers with monensin intoxication due to intraruminal boluses. **A** Focally extensive pale area of necrosis in myocardium mainly associated to papillary muscle. **B** Liver with accentuation of the lobular pattern (nutmeg appearance). **C** Focally extensive necrotizing myocarditis infiltrated by lymphocytes, macrophages, and satellite cells, in regenerative attempt with fiber disarray and fibrous connective tissue proliferation interspersed. H&E, 100×. **D** Degenerative and necrotizing myocarditis characterized by loss of cardiomyocytes, mild infiltration of satellite cells and macrophages and few remaining fibers with coagulative necrosis. H&E, 200×. **E** Diaphragm, multifocal coagulative necrosis and degeneration of myofibers, mild infiltration by macrophages and satellite cells, and a myofiber with mineralization (upper left). H&E, 200×. **F** Heart section confirming mild fibrous connective tissue (blue) replacing cardiomyocytes interspersed with neovascularization. Masson trichrome stain, 200×.

Twelve necropsies were performed on affected cattle from all 7 outbreaks. Grossly, pale multifocal streaks throughout myocardium of both ventricles were commonly observed (5/7) (Figure 2a). Also, hydrothorax (4/7), ascites (4/7), hydropericardium (4/7), hepatomegaly (enlarged liver with rounded edges, and marked tan to brown/red lobular pattern) (Figure 2b) (5/7), and diffuse enlarged lung with interlobular edema (5/7) were frequent. Other important findings were diffuse mesentery edema (3/7), abomasal edema and congestion (3/7), edema of gallbladder wall (3/7). Unusual findings included multifocal-coalescent emphysema in lung (1/7), diaphragm edema (2/7), rumen and reticulum diffuse congestion (1/7), and small and large intestine mucosal congestion with bloody or mucous content (2/7). In all necropsies, bolus devices were observed in rumen.

Tissue samples including central nervous system, heart, lung, liver, spleen, pre-stomachs, abomasum, small and large intestine, kidney, lymph nodes, and skeletal muscle were collected and fixed by immersion in 10% buffered formalin (pH 7.2) for 48 hours, then embedded in paraffin. Sections were prepared and stained with hematoxylin-eosin (H&E) for histopathological analysis. Heart sections from 6 cases and lung sections from 4 cases were stained with Masson´s trichrome for detection of collagen fiber. Microscopically, the most characteristic lesion present in all 12 necropsies was necrotizing cardiomyopathy, characterized by myocardial, multifocal-coalescent, degeneration and coagulative necrosis (hypercontraction, hypereosinophilia, fragmentation, loss of striation, absence or pyknotic nuclei) and/or infiltration of satellite cells, macrophages, and myoblasts in attempt of regeneration, and multiple areas of fibrosis (Figures 2c, 2d). Frequently, interstitial mononuclear myocarditis composed mainly by macrophages, lymphocytes and fewer plasma cells were present (10/12). Extension of mononuclear diffuse infiltrate to epicardium was present in two cases. Rarely, mineralization of myofibers (1/12) was observed. Three cases were characterized as polyphasic myocarditis. In 9 cases, myocarditis was characterized as monophasic: 4 in regenerative stage, 2 with fibrosis, 1 with degeneration and necrosis of myofibers, and 2 in an infiltrative stage. In skeletal muscle including hindlimbs in 2 cases and diaphragm in 1 case, multifocal necrosis and degeneration characterized by swollen and hypereosinophilic myofibers, loss of striations, picnotic nucleus and fragmentation of skeletal muscle cells, infiltrated by macrophages and lymphocytes, was observed; with one of them showing rarely mineralization (Figure 2e). In the remaining 10 cases no lesions were found in the sections of skeletal muscle analyzed. Multifocal to diffuse intra alveolar edema was common (5/12), with septal alveolar and interlobular thickening by macrophages, lymphocytes and hemorrhage. Also, alveolar emphysema (4/12) and atelectasis (2/12) were rarely observed. In the liver, diffuse centrilobular necrosis was the most relevant lesion (8/12). In 2 cases fatty degenerative changes were observed in hepatocyte characterized by clear intracytoplasmic vacuoles. In Masson trichrome stain, in 5 of the 6 cases, the fibrous connective tissue was evident in extensive multiple areas replacing cardiac muscle cells, interstitial, and within fibers in regeneration stage interspersed between fibroblasts, satellite cells and myoblasts (Figure 2f). In the lungs, in 2 of the 4 cases, mild to moderate perivascular fibrosis was present mainly associated to arteries.

Serum samples (n=7) were collected from affected cattle of outbreaks to determine creatine phosphokinase (CPK) enzymatic activity. Mean serum CPK was 387±318 U/l (range 89 – 998 U/l), considered moderately to highly elevated (reference value <13 U/l) (Kaneko et al., 2008).

## Discussion

The clinical history confirming administration of intraruminal monensin boluses, together with the anatomopathological findings (Ensley, 2020), strongly suggests an etiological association in all 7 outbreaks, likely due to the failure of monensin release from the boluses. In all cases steer under beef grazing systems were affected, where boluses were used to increase daily weight gain and prevent ruminal bloating during legume grazing (Grainger et al., 2008).

Boluses normally contain 10 pills of 3,2 g of monensin each, with a releasing mechanism of approximately 335 mg/d, therefore each pill should be released every 10 days (Duffield et al., 1999; Green et al., 1999). Herein in 2 of the 7 outbreaks, boluses did not release the monensin pills correctly, since all 10 pills should be released in a period of 100 days, however it occurred in 60 days. In another outbreak, 3 pills had been released over 19 days. In the remaining 2 outbreaks, the boluses contained varying amounts of residual pills. Given that they were all administered simultaneously, this suggests a failure in the release mechanism.

Another error resulting in intoxication is related to animals’ weight, as the manufacturer indicates that animals should weigh over 200 kg when boluses are administered (Elanco, 2020). However, in all 7 outbreaks of this review, at the time of administration steers weighed more than 200 kg.

Monensin intoxication through salt or premix oral administration in feed usually produces acute or per acute disease where signs appear 6 – 24hs after ingestion (Brito et al., 2020; Gonzalez et al., 2005), differing herein were clinical signs and death occurred from 30 days after bolus administration (first exposition). Fatal outcome was a common feature (Ensley, 2020), since there is no efficient treatment, and particularly myocardial lesions in monensin toxicity, as observed in all cases, are not reversible (Cooper & Valentine, 2016). In per acute monensin intoxications, gross and microscopic lesions can be mild or even absent (Ensley, 2020; Gabor & Downing, 2003), differing with these 7 outbreaks where they are evident due to progressive and prolonged exposure. Prolonged exposure to a monensin overdose, likely accounts for the extended clinical course and the eventual development of more noticeable gross myocardial lesions. Abundant liquid in abdominal and/or thoracic cavity is a gross finding in myopathies, though cardiac findings are not evident, rarely observing pale areas in myocardium (Brito et al., 2020; Nogueira et al., 2009). It is important to notice other findings indicative of chronic cardiac failure as diffuse lung edema and nutmeg liver appearance (Nogueira et al., 2009; Odriozola et al., 1999; Varga & Puschner, 2012). An important feature is that ionophore intoxication is microscopically characterized by monophasic muscular necrosis, as result of a large single toxic dose (Basaraba et al., 1999; Cooper & Valentine, 2016). Herein, polyphasic muscular lesions were observed, mainly characterized by mid-late stages of myofiber loss, fibrosis together with myonecrosis and mononuclear regenerative infiltration, probably associated with continuous mild to moderate overdosing of monensin. The fibrosis stages confirmed by Masson´s trichrome stain indicate a long-time exposure (Moxley et al., 2019; Soares et al., 2011); reinforcing that these 7 outbreaks were probably subacute to chronic, with first signs and deaths appearing 1 to 5 months after boluses administration. In addition, the perivascular fibrosis in lung sections indicates a secondary lesion originated by heart failure, reinforcing prolonged clinical course (Krafsur et al., 2019). This has been evidenced in monensin toxicosis after mild supplementation error (Brito et al., 2020). Additionally, serum CPK activity from affected steers was elevated up to 30 times, associated to muscular damage due to monensin overdose (Cooper & Valentine, 2016).

## Conclusion

Monensin toxicosis is a frequent pathology in cattle due to oral dosing errors. However, to our knowledge there are no reports of toxicosis after failure of intraruminal boluses in grazing cattle as herein. Though these episodes are rare it is important to describe the occurrence of cases when release bolus mechanism fails, leading to subacute to chronic clinicopathological presentations with polyphasic muscular histopathological findings.
